# InterVFast—effectiveness and acceptance of intermittent fasting in cardiac rehabilitation patients: study protocol of a randomized controlled trial

**DOI:** 10.1186/s13063-023-07843-7

**Published:** 2024-01-09

**Authors:** Claudia Pieper, Florian Tim Hitesh Kaistha, Sarah Schroeer, Maria Borgert, Andreas Michalsen, Wolfgang Mayer-Berger

**Affiliations:** 1 Institute for Medical Informatics, Biometry and Epidemiology (IMIBE), University Hospital Essen, University of Duisburg-Essen, Hufelandstr. 55, 45147 Essen, Germany; 2 Department of Internal and Integrative Medicine, Immanuel Hospital Berlin, Berlin, Germany; 3 Centre for Cardiovascular Rehabilitation, 42799 Leichlingen, Germany

**Keywords:** Intermittent fasting, Weight loss, Effectiveness, Acceptance, Randomized controlled trial, Rehabilitation, Prevention

## Abstract

**Background:**

Research on intermittent fasting has shown that it can improve a variety of health outcomes, including blood sugar control, blood lipid levels and blood pressure. Only few studies document longer periods of fasting, especially in rehabilitation participants. Cardiac inpatient rehabilitation follows a multidisciplinary approach including change of health behaviour to reduce patients’ risk of future cardiovascular events. To date, evidence suggests that intermittent fasting can be an effective way to improve health and well-being, but more research is needed to fully understand its long-term effects and factors that promote the implementation. Therefore, the aim of the ongoing InterVFast trial is to investigate the effectiveness of intermittent fasting amongst cardiac rehabilitation patients after 4-week inpatient rehabilitation as well as 3 and 12 months subsequently including patients’ perspective.

**Methods:**

This single-centre randomized controlled trial evaluates the effectiveness of the InterVFast intervention in weight loss (primary outcome). We also examine patients’ acceptance and the effect on relevant outcomes as blood glucose and triglyceride levels, cholesterol and high-sensitivity C-reactive protein. Weight, blood samples and clinical data are collected as part of the initial and final examination during inpatient rehabilitation. During inpatient rehabilitation, participants daily note fasting intervals and meals eaten as well as practicability in a fasting diary. In addition, interviews about perceived advantages and disadvantages and acceptance are carried out with the participants in the IG. A standardized follow-up examination (weight, blood samples) will be carried out by the family doctor after 3 and 12 months (t2 and t3).

**Discussion:**

Compared to other weight-loss intervention studies, our study addresses patients with coronary heart disease and includes patients’ acceptance as well as long-term maintenance. It is hypothesized that participation in the InterVfast intervention will improve relevant health outcomes in a sample of cardiac rehabilitation patients and thus constitute a behavioural prevention strategy to reduce the risk of future cardiac events and improve overall health and quality of life.

**Trial registration:**

ClinicalTrials.gov DRKS00023983. Registered on February 17, 2022.

**Supplementary Information:**

The online version contains supplementary material available at 10.1186/s13063-023-07843-7.

## Administrative information

**Table:** 

Title	InterVFast –Effectiveness and acceptance of intermittent fasting in cardiac rehabilitation patients – Study protocol of a randomised controlled trial
Trial registration {2a and 2b}.	German Clinical Trials Register, DRKS00023983. Registered 17 February 2022, https://www.drks.de.
Protocol version {3}	25-01-2021, version 21007-01.2
Funding {4}	Rehabilitation research network (refonet) of the German Pension Insurance Rhineland, Grant-nr. 21007
Author details {5a}	Claudia Pieper1, Sarah Schroeer1, Maria Borgert1, Andreas Michalsen2, Wolfgang Mayer-Berger3. 1 Institute for Medical Informatics, Biometry and Epidemiology (IMIBE), University Hospital Essen, University of Duisburg-Essen, Hufelandstr. 55, 45147 Essen, Germany 2 Department of Internal and Integrative Medicine, Immanuel Hospital Berlin, Berlin, Germany 3 Centre for Cardiovascular Rehabilitation, 42799 Leichlingen, Germany
Name and contact information for the trial sponsor {5b}	Rehabilitation research network of the German Pension Insurance Rhineland (german: Rehabilitationsforschungsnetzwerk der Deutschen Rentenversicherung Rheinland‘ (refonet)) Adress: Hochstraße 13–19, 53474 Bad Neuenahr-Ahrweiler Phone: +49 2641 9062-, E-Mail: service@refonet.de
Role of sponsor {5c}	None. The prinicipal investigator has ultimate authority over any research activities including publication.

## Introduction

### Background and rationale

#### Cardiac rehabilitation

Cardiac rehabilitation is a programme for patients who experienced heart attack, heart surgery or other cardiovascular events [1, 18, 20]. Cardiac inpatient rehabilitation typically combines exercise, education and counselling in a multidisciplinary approach to reduce patients’ risk of future cardiovascular events [12, 18]. The goals of cardiac rehabilitation include improving cardiovascular health, reducing symptoms and helping patients make lifestyle changes that promote participation in working life [6, 18]. Although adherence to lifestyle interventions is challenging in patients with cardiovascular disease, we previously showed that a low-threshold intervention can improve patients’ motivation to continue to practise home based [20].

#### Intermittent fasting

Intermittent fasting (IF) is an eating pattern that involves alternating periods of fasting and eating [2, 5, 15]. For instance, 16:8-intermittent fasting involves fasting for 16 h a day and eating during the remaining 8 h. While a 16:8 intermittent fasting plan does not specify which foods to eat and avoid, it is beneficial to focus on healthful eating and to limit or avoid highly processed foods [21].

The scientific background of intermittent fasting is based on the understanding of how the body processes food and the effects of caloric restriction on health and longevity.

When we eat, the body breaks down the food into glucose, which is used for energy, and insulin, which helps regulate blood sugar levels. During periods of fasting, the body switches from using glucose as its primary source of energy to using stored fat [4, 11]. This metabolic shift has been linked to a range of health benefits, including improved insulin sensitivity, reduced inflammation and increased autophagy, the process by which the body breaks down and recycles damaged cells [4, 7, 11].

Research on intermittent fasting has shown that it can improve a variety of health outcomes in animal-studies. Wan et al. found that intermittent fasting increases lifespan and improves overall health. Mice that fasted every other day had a longer lifespan and were less likely to develop age-related diseases [26].

Human studies indicate that intermittent fasting improves blood sugar control and blood lipid levels and reduces the risk of type 2 diabetes [22, 25]. Some studies also suggest that intermittent fasting helps to promote weight loss and improve cognitive function [22, 24, 25].

Additionally, intermittent fasting has a range of potential health benefits, including reduced inflammation. Participants who follow an intermittent fasting diet are more likely to lose weight and have greater improvements in insulin sensitivity than those who follow a traditional calorie-restricted diet [13, 16]. Nevertheless, Ostendorf et al. emphasize that more research is needed to fully understand the long-term effects of intermittent fasting [16].

### Objectives

To date, evidence suggests that intermittent fasting can be an effective way to improve health and well-being, but more research is needed to fully understand its long-term effects [16, 22]. Only few studies document longer periods of fasting in high-risk person such as rehabilitation patients. Therefore, the aim of the ongoing InterVFast trial is to investigate the effectiveness of intermittent fasting amongst cardiac rehabilitation patients after 4-week inpatient rehabilitation as well as 3 and 12 months subsequently including patients’ perspective.

### Trial design

Our single-centre randomized controlled trial (RCT) evaluates the effectiveness of the InterVFast intervention regarding short-term as well as long-term weight loss (primary outcome). We also examine patients’ acceptance and the effect on relevant outcomes as blood glucose and triglyceride levels, cholesterol and high-sensitivity C-reactive protein.

## Methods: participants, interventions and outcomes

### Study setting

The study is conducted in the centre for cardiovascular rehabilitation in the city of Leichlingen, North Rhine-Westphalia, Germany. While Leichlingen is a more rural town, the catchment area of the centre is the largest metropolitan area of Germany, a population of over 8 million people within a 50-km radius, including big cities like Cologne (population: 1 million) and Duesseldorf (population: 600.000), but also very rural areas.

### Eligibility criteria

The inclusion criteria for this study are the following: male sex and age between 18 and 60 years, a diagnosis of coronary heart disease (CHD) and a body mass index (BMI) between at least 27 kg/m^2^ and 38.9 kg/m^2^.

The study exclusion criteria are as follows: a *NYHA (New York Heart Association Classification)* Functional Class IV, severe obesity (BMI > 39 kg/m^2^), oncological diseases, gastric or duodenal ulcers and insulin dependent diabetes. Patients with an intellectual disability, a current eating disorder or other psychiatric disorders (psychotic disorders, bipolar disorder, substance dependence, or anxiety and depressive disorders) are excluded.

However, we decided to exclude women because of the following reasons: first, some evidence indicates that intermittent fasting may have negative effects on reproduction and blood sugar levels in some women [3, 8, 17]. In all likelihood, such an intervention would produce different results not only in men and women but also in pre- and postmenopausal women [19, 23].

Secondly, female patients are generally underrepresented in cardiac rehabilitation (female-to-male ratio 1:4)^1^ as well as in our study centre. In 2022, only 15% of all patients with CD were female. Therefore, to enroll an adequate number of patients in reasonable time, we solely recruited male patients. Over and beyond, only few studies on lifestyle changing interventions such as intermittent fasting address men. Since men are underrepresented relative to women in current research concerning intermittent fasting, this trial still enriches the existing state of research [9, 10].

### Who will take informed consent?

Two qualified and appropriately trained members of the research team are responsible for obtaining consent. Informed consent involves giving information to the potential participant and clarifying the information. In obtaining and documenting informed consent, the research team complies with good clinical practice (GCP) and with the ethical principles of the Declaration of Helsinki [27].

### Additional consent provisions for collection and use of participant data and biological specimens

No additional consent is provided for collection and use of participant data who withdraw; this study does not involve collecting biological specimens for storage.

### Interventions

#### Explanation for the choice of comparators

The primary outcome of this study is difference in mean weight loss between baseline and end of inpatient rehabilitation after 4 weeks (short-term effectiveness). This outcome has been selected as the primary outcome, because intermittent fasting addresses weight as a main risk factor for cardiovascular disease.

#### Intervention description

The InterVFast intervention was designed by a cooperation of researchers and clinicians from the fields of epidemiology, cardiology and rehabilitation medicine and nutritional advice. The specific contents of the intervention are as follows:

##### 16:8-intermittent fasting

The intervention consists of a 16:8-intermittent fasting programme during the 4-week inpatient rehabilitation. 16:8-intermittent fasting involves fasting for 16 h a day and consuming all calories during the remaining 8 h. For example, a participant starts eating at 11:00 am and stops eating at 7:00 pm. The fasting window is individually adapted to the wishes and needs of the participants.

The fasting programme includes an introduction to the concept of fasting by a nutritionist providing information on the health benefits, practical implementation and fasting tips. Participants in the IG document periods of fasting and meals eaten in a paper-based fasting diary.

A fasting consultation is provided by a trained nutritionist twice a week to answer questions and to track individual progress. Purpose of the consultation can be wants, requests, intentions, preferences or expectations. Besides that, the inpatient rehabilitation programme is carried out as usual.

##### Standard treatment

During 4-week rehabilitation period, the CG receives standard treatment. Regarding eating behaviour, the standard programme includes “healthy-eating” training (2 h), shopping tips for healthy eating (1 h), a teaching kitchen of 3 h, and daily food and beverage buffet training (30 min).

#### Criteria for discontinuing or modifying allocated interventions

Criteria for discontinuing or modifying allocated interventions are any changes in the patient’s condition that justify the discontinuation of treatment in the clinician’s opinion, and participant withdraws consent and confirmation of non-eligibility after registration.

#### Strategies to improve adherence to interventions

During inpatient rehabilitation, a trained nutritionist held regular fasting visits to improve adherence to the fasting programme.

#### Relevant concomitant care permitted or prohibited during the trial

There are no restrictions regarding concomitant care during the trial.

#### Provisions for posttrial care

Participants are able to contact the research team during the study period as well as after the end of participation.

### Outcomes

The primary outcome of this study is difference in weight loss between baseline and end of inpatient rehabilitation after 4 weeks (short-term effectiveness). Secondary outcomes are difference in weight loss between baseline and 3 and 12 months after inpatient rehabilitation (long-term effectiveness), changes in blood pressure, fasting blood glucose and lipids and changes in diet as well as patients’ acceptance.

Weight, blood samples and clinical data are routinely collected as part of the initial and final examination. During inpatient rehabilitation, participants note daily periods of fasting as well as practicability (rating scale from 0 — very bad to 5 — very easy) in a fasting diary. They record all food and drinks, type of meal (breakfast, lunch, dinner, or snack), type of food and portion size. In addition, we carry out interviews about perceived advantages and disadvantages and acceptance with all participants in the IG.

After initial examination at admission (baseline, t0), three follow-up times are provided: final examination at the end of inpatient rehabilitation after 4 weeks (t1), as well as two follow-up times after inpatient rehabilitation after 3 and 12 months. The World Health Organization-Five Well-Being Index (WHO-5) is used for self-reported measure of current mental wellbeing at t0 and t1. A standardized follow-up examination (weight, blood samples) will be carried out by the family doctor at t2 and t3 for valid information. A financial incentive will be paid to family doctors for examination and data transfer.

Sociodemographic characteristics and comorbidities at baseline will be collected as moderating variables. Stable covariables (e.g. sociodemographic characteristics) will be collected only at baseline, while changeable variables will be measured at baseline and follow-up.

#### Participant timeline

**Fig. 1:**
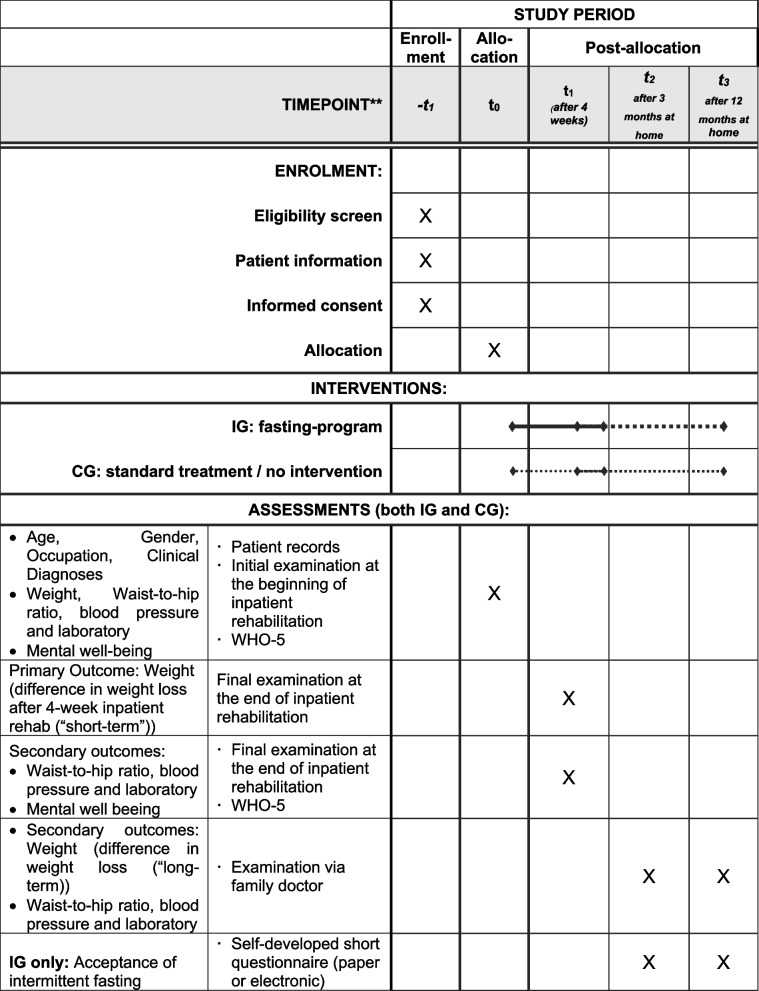
Schedule of enrolment, intervention and assessments

Eligibility screening took place at initial admission examination (t0) involving medical history review, physical exams and laboratory tests. Figure 1 shows the schedule of enrolment, intervention and assessments.

Patients who met the inclusion criteria were invited to provide study information at the beginning of the inpatient rehabilitation in a personal conversation. We explained the study’s purpose, procedures and potential risks and benefits to the patient. Each patient received appropriate written participant information and an informed consent form.

#### Sample size

A before-and-after comparison between the intervention group (IG) and the control group (CG) will be used for the evaluation. Follow-up values were assessed at t1, t2 and t3. Regarding the primary outcome, the intervention is considered effective if mean difference in weight loss after 4 weeks (t1) in the IG is at least 2% higher than in the CG compared to baseline (t0). The standard deviation of mean difference is estimated at 3.5 kg. The significance level should be 5%. In order to demonstrate difference in weight with a power of 80%, a two-tailed *t*-test requires a total of 100 patients (50 per group).

#### Recruitment

Based on an estimated participation rate of 20%, a period of 6 months was planned for the implementation of the intervention and patient enrollment.

### Assignment of interventions: allocation

#### Sequence generation

As soon as the patient gave written consent, randomization was performed. Randomization took place at individual level, i.e. every single patient was assigned either to the IG or to the CG. A statistician who is not involved in the study generated the randomization list using computer-generated random numbers.

#### Concealment mechanism

The research team member recruiting the patients contacted the randomization office by phone or secure computer after the patient was enrolled in the study (central randomization).

#### Implementation

After eligibility screening took place at initial admission examination (t0) involving medical history review, physical exams and laboratory tests, two trained members of the research team enrolled patients. To minimize the effect of bias, the random allocation sequence remained concealed from those enrolling patients into the study.

#### Assignment of interventions: blinding

Considering the nature of the intervention, participants and therapists cannot be blinded. So, the design of the study is open label; therefore, unblinding will not occur.

The statistician was not blinded, because, to our mind, the statistician should have a good knowledge of the topic of cardiovascular rehabilitation and the intervention. The statistician was not involved in the intervention and data collection.

### Data collection and management

#### Plans for assessment and collection of outcomes

The primary outcome of this study is difference in weight loss between baseline and end of inpatient rehabilitation after 4 weeks.

Secondary outcomes are difference in weight loss between baseline and 3 and 12 months after inpatient rehabilitation, changes in blood pressure, fasting blood glucose and lipids and changes in diet as well as patients’ acceptance.

Weight, blood samples and clinical data are routinely collected as part of the initial and final examination. During inpatient rehabilitation, participants note daily periods of fasting as well as practicability (rating scale from 0 — very bad to 5 — very easy) in a fasting diary. They record all food and drinks, type of meal (breakfast, lunch, dinner, or snack), type of food and portion size.

After initial examination at admission (baseline, t0), three follow-up times are provided: final examination at the end of inpatient rehabilitation after 4 weeks (t1), as well as two follow-up times after inpatient rehabilitation after 3 and 12 months. The World Health Organization-Five Well-Being Index (WHO-5) is used for self-reported measure of current mental wellbeing at t0 and t1. A standardized follow-up examination (weight, blood samples) will be carried out by the family doctor at t2 and t3 for valid information.

#### Plans to promote participant retention and complete follow-up

To assure participant retention and completion of follow-up for t2 und t3, we send a reminder about completing the survey to participants who have not yet participated in the follow-up evaluation. A financial incentive will be paid to family doctors for follow-up examination and data transfer.

All baseline data is collected also for participants who discontinue or deviate from intervention protocols.

#### Data management

We perform double data entry and range checks for data values. Data quality management also includes data cleansing (completeness, consistency, duplicates) and validation of data against standard statistical measures. Co-investigator meetings were held monthly during the recruitment phase and then quarterly.

#### Onsite-research team

The members of the onsite-research team facilitate and coordinate the study activities in the rehabilitation centre. They provide intervention measures and collect data as required by the protocol. They provide information for the PI and the team members who are responsible for administration, randomization and analysis.

#### Confidentiality

For the statistical analysis, personalized data is documented only in the rehabilitation centre. Solely, pseudonymized data is sent to the Institute for Medical Informatics, Biometry and Epidemiology (IMIBE) for analysis. All employees are familiar with quality-assured data collection, data monitoring and documentation.

#### Plans for collection, laboratory evaluation and storage of biological specimens for genetic or molecular analysis in this trial/future use

This study does not involve collecting and storing biological specimens for storage in the current trials and for future use in ancillary studies.

### Patient/public involvement

We conduct structured patient interviews to evaluate the patient’s experience with the intervention. Public involvement takes place in the course of dissemination of generally understandable study results.

### Statistical methods

#### Statistical methods for primary and secondary outcomes

The statistical evaluation is carried out using the statistical software SPSS (IBM SPSS Statistics, version 29). The confirmatory analysis to measure the effect of the intervention will be carried out as an intention-to-treat analysis, i.e. all participants who are randomized are included in the statistical analysis and analysed according to the group they were originally assigned. Additional analyses will focus on secondary outcomes as well as associations between independent and dependent variables.

Fasting diaries will be analysed using in-house nutrition information. The mean daily caloric intakes will be computed.

The WHO-5 uses a 6-point Likert scale ranging from 0 = “at no time” to 5 = “all of the time”. The sum of these answers is multiplied by 4; scores of 50 or lower suggest that participants might suffer from depression.

Between-group differences at baseline will be tested using analysis of variance (ANOVA). To answer the primary research question, the mean difference in weight loss between the two groups is compared using analysis of variance (ANOVA), a two-tailed *t*-test, or Spearman’s correlation coefficient, respectively. Chi-square tests are used to compare proportions. All continuous outcomes will be modelled using linear regression. We will analyse the effect of fasting while taking into account periods of fasting and calorie intake from the fasting diaries. All statistical tests assume a two-sided significance level of 0.05.

Guided interviews are transcribed and coded by two independent researchers along an open coding scheme using qualitative content analysis by Mayring [14] via the software MAXQDA (VERBI GmbH, Berlin, Germany). Qualitative content analysis aims at classifying qualitative data into categories of similar connotation.

#### Interim analyses

This randomized trial does not incorporate interim analyses to stop the study for futility. Coming from experience, we neither assume not to reach the full planned sample size nor that early data would suggest that an important treatment effect is unlikely to be found, even if the study continues to its full planned sample size.

#### Methods for additional analyses

We will analyse the effect of sociodemographic and clinical variables on the primary outcome as well as the effect of periods of fasting and calorie intake. Additionally, stratified analysis will be performed for those who completed t2 and t3 follow-up. All statistical tests assume a two-sided significance level of 0.05.

#### Methods in analysis to handle protocol non-adherence and any statistical methods to handle missing data

We use the ITT approach; all randomized patients are included in the analysis, based on the groups to which they were initially randomly assigned. To assess the bias of your data due to non-response, we will perform a non-response bias analysis.

#### Plans to give access to the full protocol, participant level-data and statistical code

There are no plans to grant access to full protocol, participant-level dataset or statistical code.

### Oversight and monitoring

#### Composition of the coordinating centre and trial steering committee

All investigators are steering committee members, including an external independent expert member. Investigator meetings are held regularly to monitor and supervise the progress of the study towards its short-term and overall objectives and adherence to the protocol.

#### Composition of the data monitoring committee, its role and reporting structure

We did not implement a special data monitoring committee, because the members of the data research team working on data management are well versed. We performed internal monitoring to verify data within the constraints of the regulatory requirements two times during the data collection phase. A data monitoring committee was not considered as this was a low-risk intervention co-investigator meetings including auditing trial conduct which was held monthly during the recruitment phase and then quarterly.

#### Adverse event reporting and harms

It is the responsibility of the investigators to report serious adverse events. An adverse event is defined as any untoward medical occurrence in a subject without regard to the possibility of a causal relationship. Adverse events will be collected after the subject has provided consent and enrolled in the study. All adverse events occurring after entry into the study and until rehabilitation discharge will be recorded.

Based on our experience with comparable trials, no adverse events related to the intervention are expected.

#### Frequency and plans for auditing trial conduct

To assess and assure the reliability and integrity against all relevant written standards, a systematic internal examination is conducted twice a year (three times in total). We will verify the following variables for all patients: initials, date of birth, sex, signed informed consent, eligibility criteria, date of randomization, treatment assignment, adverse events and endpoints. The process is independent from the funder.

#### Plans for communicating important protocol amendments to relevant parties

Important protocol modification changes will be reported to the ethical committee and other relevant parties.

#### Dissemination plans

We developed a publication strategy to select appropriate content, formats and audiences. The trial results will be reported to participants and the public in a public-centred research report. Healthcare professionals and the scientific community will be informed via peer-reviewed journal publications and conference proceedings.

According to the guiding principle of the PI’s affiliation, research publications are not influenced or controlled by funders or investigators. We will publish both positive and negative outcomes in an equitable manner.

## Discussion

For the first time, the InterVFast intervention systematically investigates shot term as well as long-term effects of intermittent fasting on weight loss and cardiovascular health outcomes within a cardiac inpatient rehabilitation sample. Compared to other weight-loss intervention studies, our approach addresses patients with CHD and focuses on patients’ acceptance and long-term maintenance. However, since men are underrepresented relative to women in current research concerning lifestyle changing interventions, this trial enriches the existing state of research.

Yet — if proven effective — the findings of this study could strengthen the idea that intermittent fasting may be a practicable intervention. Beyond the cardiac rehabilitation context, the InterVFast intervention could be model for other chronic conditions and medical settings. Future research should explore the effectiveness of intermittent fasting in both premenopausal and postmenopausal women with CHD and provide evidence of the effectiveness of maintenance strategies considering factors that help to promote patients’ motivation.

### Trial status

The trial is ongoing. The recruitment of participants started on 23 March 2022 and was completed on 14 September 2022. A total of 278 of 352 patients screened met inclusion criteria, and 120 were willing to participate and gave informed consent.

- Recruitment status and baseline evaluation: Completed
- Follow-up status t1: Completed
- Follow-up status t2 and t3: Ongoing

### Supplementary Information

**Additional file 1**: SPIRIT 2013 Checklist: Recommended items to address in a clinical trial protocol and related documents.

## Data Availability

There are no plans to grant access to full protocol, participant-level dataset, or statistical code.

## Abbreviations

ANOVA: Analysis of variance
BMI: Body mass index
CHD: Coronary heart disease
IMIBE: Institute for Medical Informatics, Biometry and Epidemiology
IG: Intervention group
CG: Control group
NYHA: New York Heart Association Classification
RCT: Randomized controlled trial
WHO-5: World Health Organization-Five Well-Being Index

## Footnotes

1 www.deutsche-rentenversicherung.de/DRV/DE/Experten/Zahlen-und-Fakten/Statistiken-und-Berichte/statistiken-und-berichte_node.html
