# Supplementary material for: InterVFast—effectiveness and acceptance of intermittent fasting in cardiac rehabilitation patients: study protocol of a randomized controlled trial
Source: Trials. 2024 Jan 9;25:32. doi: 10.1186/s13063-023-07843-7 (PMC10775558; doi:10.1186/s13063-023-07843-7)
Supplement: Supplementary file 1 — Additional file 1: SPIRIT 2013 Checklist: Recommended items to address in a clinical trial protocol and related documents. [file 13063_2023_7843_MOESM1_ESM.doc]

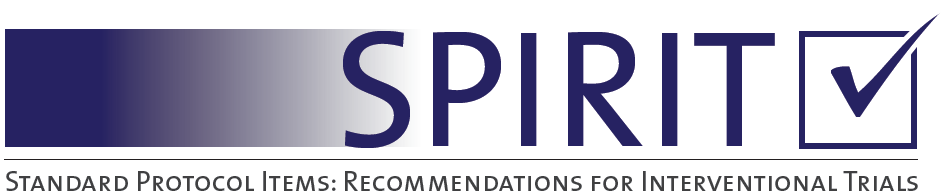


SPIRIT 2013 Checklist: Recommended items to address in a clinical trial protocol and related documents*

| Section/item | ItemNo | Description |
| --- | --- | --- |
| **Administrative information (modifications are marked)** | | |
| Title | 1 | InterVFast – ~~Long-term~~ Effectiveness and acceptance of intermittent fasting in cardiac rehabilitation patients – Study protocol of a randomised controlled trial |
| Trial registration | 2a | German Clinical Trials Register, DRKS00023983. Registered 17 February 2022, https://www.drks.de. |
|  | 2b | Contact for Public / Scientific Queries: Claudia Pieper, Institute for Medical Informatics, Biometry and Epidemiology (IMIBE), University Hospital Essen, Hufelandstr. 55, 45147 Essen, Germany, Claudia.pieper@uk-essen.de |
| Protocol version | 3 | 25-01-2021, version 21007-01.2 |
| Funding | 4 | Funder: Rehabilitation research network (refonet) of the German Pension Insurance Rhineland, Grant-nr. 21007 |
| Roles and responsibilities | 5a | Claudia Pieper^1^, Sarah Schroeer^1^, Maria Borgert^1^, Andreas Michalsen^2^, Wolfgang Mayer-Berger^3^.  1 Institute for Medical Informatics, Biometry and Epidemiology (IMIBE), University Hospital Essen, University of Duisburg-Essen, Hufelandstr. 55, 45147 Essen, Germany  2 Department of Internal and Integrative Medicine, Immanuel Hospital Berlin, Berlin, Germany  3 Centre for Cardiovascular Rehabilitation, 42799 Leichlingen, Germany  CP: principal investigator  SaS: statistics  MB: intervention design  WMB: clinical expertise  AM: scientific advice on intermittent fasting |
|  | 5b | Primary Sponsor: University Hospital Essen (represented by the executive committee), Hufelandstr. 55, 45147 Essen, Germany |
|  | 5c | Role of funders: none  The prinicipal investigator has ultimate authority over any research activities including publication |
|  | 5d | Composition, roles, and responsibilities:  All investigators are steering committee members, including an external independent expert member. Investigator meetings have been held quarterly to monitor and supervise the progress of the study towards its short-term and overall objectives and adherence to the protocol. |
| Introduction |  |  |
| Background and rationale | 6a | Research on intermittent fasting has shown that it can improve a variety of health outcomes, including blood sugar control, blood lipid levels and blood pressure. Only few studies document longer periods of fasting, especially in rehabilitation participants. Cardiac inpatient rehabilitation follows a multidisciplinary approach including change of health behaviour to reduce patients` risk of future cardiovascular events. To date, evidence suggests that intermittent fasting can be an effective way to improve health and well-being, but more research is needed to fully understand its long-term effects and factors that promote the implementation. Therefore, the aim of the ongoing InterVFast trial is to investigate the effectiveness of intermittent fasting amongst cardiac rehabilitation patients in the long term including patients’ perspective. |
|  | 6b | Active comparator: intermittent fasting-program in inpatient rehabilitation (4 weeks) vs. standard treatment |
| Objectives | 7 | Regarding the primary outcome, the intervention is considered effective if mean difference in weight loss after 4 weeks (t1) in the IG is at least 2% higher than in the CG compared to baseline (t0). |
| Trial design | 8 | This single-center randomised controlled trial evaluates the effectiveness of the InterVFast intervention regarding short-term as well as long-term weight loss (primary outcome). We also examine patients’ acceptance and the effect on relevant outcomes as blood glucose and triglyceride levels, cholesterol and High-sensitivity C-reactive protein.  Weight, blood samples and clinical data are collected as part of the initial and final examination during inpatient rehabilitation. During inpatient rehabilitation, participants daily note fasting intervals and meals eaten as well as practicability in a fasting diary. In addition, interviews about perceived advantages and disadvantages and acceptance are carried out with the participants in the IG.  A standardized follow-up examination (weight, blood samples) will be carried out by the family doctor after three and twelve months (t2 and t3).  A before-and-after comparison between the IG and the CG will be used for the evaluation. The confirmatory analysis to measure the effect of the intervention will be carried out as an intention-to-treat analysis, i.e. all participants who are randomized are included in the statistical analysis and analysed according to the group they were originally assigned. |
| Methods: Participants, interventions, and outcomes | | |
| Study setting | 9 | The study is conducted in the centre for cardiovascular rehabilitation in the city of Leichlingen, North Rhine-Westphalia, Germany. While Leichlingen is a more rural town, the catchment area of the centre is the largest metropolitan area of Germany, a population of over 8 million people within a 50 km radius, including big cities like Cologne (population: 1 million) and Duesseldorf (population: 600.000), but also very rural areas. |
| Eligibility criteria | 10 | The inclusion criteria for this study are the following: male sex and age between 18 and 60 years, a diagnosis of coronary heart disease (CHD) and a body mass index (BMI) between at least 27 kg/m2 and 38.9 kg/m2.  The study exclusion criteria are: a *NYHA (New York Heart Association Classification*) Functional Class IV, severe obesity (BMI > 39 kg/m2), oncological diseases, gastric or duodenal ulcers and insulin dependent diabetes. Patients with an intellectual disability, a current eating disorder or other psychiatric disorders (psychotic disorders, bipolar disorder, substance dependence, or anxiety and depressive disorders) are excluded. |
| Interventions | 11a | The specific contents of the intermittent fasting-intervention are as follows:  16:8-intermittent fasting  The intervention consists of a 16:8-intermittent fasting program during the four-week inpatient rehabilitation. 16:8-intermittent fasting involves fasting for 16 hours a day and consuming all calories during the remaining 8 hours. For example, a participant starts eating at 11:00 am and stops eating at 7:00 pm. The fasting window is individually adapted to the wishes and needs of the participants.  The fasting program includes an introduction to the concept of fasting by a nutritionist providing information on the health benefits, practical implementation and fasting tips. Participants in the IG document periods of fasting and meals eaten in a paper-based fasting diary.  A fasting consultation is provided by a trained nutritionist twice a week to answer questions and to track individual progress. |
|  | 11b | Criteria for discontinuing or modifying allocated interventions:   - Any change in the patient's condition that justifies the discontinuation of treatment in the clinician's opinion - Participant withdraws consent - Non-eligibility is confirmed after registration. |
|  | 11c | Regular fasting visits  A standardized follow-up examination (weight, blood samples) will be carried out by the family doctor at t2 and t3 for valid information. |
|  | 11d | There are no restrictions regarding concomitant care during the trial. |
| Outcomes | 12 | The primary outcome of this study is difference in weight loss between baseline and end of inpatient rehabilitation after 4 weeks. This outcome has been selected as the primary outcome, because intermittent fasting addresses weight as a main risk factor for cardiovascular disease. Secondary outcomes are difference in weight loss between baseline and 3 and twelve 12 months after inpatient rehabilitation, changes in blood pressure, fasting blood glucose and lipids, and changes in diet as well as patients’ acceptance. |
| Participant timeline | 13 | Time schedule of enrolment, interventions (including any run-ins and washouts), assessments, and visits for participants. A schematic diagram is highly recommended (*see Figure 1*) |
| Sample size | 14 | Regarding the primary outcome, the intervention is considered effective if mean difference in weight loss after 4 weeks (t1) in the IG is at least 2% higher than in the CG compared to baseline (t0). The standard deviation of mean difference is estimated at 3.5kg. The significance level should be 5%. In order to demonstrate difference in weight with a power of 80%, a two-tailed t-test requires a total of 100 patients (50 per group). |
| Recruitment | 15 | Based on experience, the estimated participation rate is 20%. Therefore a period of six months was planned for the implementation of the intervention and patient enrollment. |
| **Methods: Assignment of interventions (for controlled trials)** | | |
| Allocation: |  |  |
| Sequence generation | 16a | Method of generating the allocation sequence: computer-generated random numbers. The randomisation list was generated by a statistician who was not involved in the study. |
| Allocation concealment mechanism | 16b | Central randomization. The individual recruiting the patient contacted the randomization-office by phone or secure computer after the patient was enrolled |
| Implementation | 16c | Eligibility screening took place at initial admission examination (t0) involving medical history review, physical exams and laboratory tests.  Two trained members of the research team enrolled patients.  To minimize the effect of bias, the random allocation sequence remained concealed from those enrolling patients into the study. |
| Blinding (masking) | 17a | The statistician was not blinded, because, to our mind, the statistician should have a good knowledge of the topic of cardiovascular rehabilitation and the intervention. The statistician was not involved in the intervention and data collection. |
|  | 17b | Considering the nature of the intervention, participants and therapists cannot be blinded, so the design of the study is open label and unblinding will not occur. |
| **Methods: Data collection, management, and analysis** | | |
| Data collection methods | 18a | The primary outcome of this study is difference in weight loss between baseline and end of inpatient rehabilitation after 4 weeks (short term effectiveness).  Secondary outcomes are difference in weight loss between baseline and 3 and twelve 12 months after inpatient rehabilitation (long term effectiveness), changes in blood pressure, fasting blood glucose and lipids, and changes in diet as well as patients’ acceptance.  Weight, blood samples and clinical data are routinely collected as part of the initial and final examination. During inpatient rehabilitation, pparticipants note daily periods of fasting as well as practicability (rating scale from 0 - very bad to 5 - very easy) in a fasting diary. They record all food and drinks, type of meal (breakfast, lunch, dinner, or snack), type of food and portion size.  After initial examination at admission (baseline, t0), three follow-up times are provided: final examination at the end of inpatient rehabilitation after 4 weeks (t1), as well as two follow-up times after inpatient rehabilitation after 3 and 12 months. The World Health Organisation-Five Well-Being Index (WHO-5) is used for self-reported measure of current mental wellbeing at t0 and t1. A standardized follow-up examination (weight, blood samples) will be carried out by the family doctor at t2 and t3 for valid information.  (eg, duplicate measurements, training of assessors) and a description  Data collection forms are available on request.  Data quality: data cleansing (completeness, consistency, duplicates), Validation of data against standard statistical measures |
|  | 18b | Participant retention and complete follow-up for t2 und t3: regular recall is performed and documented  All baseline data is collected for participants who discontinue or deviate from intervention protocols. |
| Data management | 19 | For the statistical analysis, collected data is documented in the rehabilitation center. Pseudonymised data is sent to the Institute for Medical Informatics, Biometry and Epidemiology (IMIBE) for analysis. All employees are familiar with quality-assured data collection, data monitoring and documentation. |
| Statistical methods | 20a | The statistical evaluation is carried out using the statistical software SPSS (IBM SPSS Statistics, version 29). The confirmatory analysis to measure the effect of the intervention will be carried out as an intention-to-treat analysis, i.e. all participants who are randomized are included in the statistical analysis and analysed according to the group they were originally assigned. Additional analyses will focus on secondary outcomes as well as associations between independent and dependent variables.  Fasting diaries will be analysed using in-house nutrition information. The mean daily caloric intakes will be computed.  The WHO-5 uses a six-point Likert scale ranging from 0 = “at no time” to 5 = “all of the time”. The sum of these answers is multiplied by 4; scores of 50 or lower suggest that participants might suffer from depression.  Between-group differences at baseline will be tested using Analysis of Variance (ANOVA). To answer the primary research question, the mean difference in weight loss between the two groups is compared using Analysis of Variance (ANOVA), a two-tailed t-test or Spearman's correlation coefficient, respectively. Chi-square tests are used to compare proportions. |
|  | 20b | We will analyse the effect of fasting, while taking into account periods of fasting and calorie intake from the fasting diaries. All statistical tests assume a two-sided significance level of 0.05. |
|  | 20c | Following the ITT approach, all randomized patients are included in the analysis, based on the groups to which they were initially randomly assigned.  We will perform stratified analyses of nonresponders, t2- and t3-responders. |
| **Methods: Monitoring** | | |
| Data monitoring | 21a | A DMC is not needed, because of quality assured data management within the project team. Investigator meetings are held regularly.  We will conduct monitoring two times by an external committee. |
|  | 21b | This randomised trial does not incorporate interim analyses to stop the study for futility. Coming from experience, we did not assume that early data would suggest that an important treatment effect is unlikely to be found, even if the study were to continue to its full planned sample size. |
| Harms | 22 | It is the responsibility of the investigators to report serious adverse events. An adverse event is defined as any untoward medical occurrence in a subject without regard to the possibility of a causal relationship. Adverse events will be collected after the subject has provided consent and enrolled in the study. All adverse events occurring after entry into the study and until rehabilitation discharge will be recorded.  Based on our experience with comparable trials, no adverse events are expected. |
| Auditing | 23 | To assess and assure the reliability and integrity against all relevant written standards a systematic internal examination is conducted twice a year (3 times in total). We will verify the following variables for all patients: initials, date of birth, sex, signed informed consent, eligibility criteria, date of randomization, treatment assignment, adverse events, and endpoints.  The process is independent from investigators and the sponsor.  A Data Monitoring Committee was not considered as this was a low-risk intervention Co-investigator meetings including auditing trial conduct were held monthly during the recruitment phase and then quarterly. |
| Ethics and dissemination | | |
| Research ethics approval | 24 | The study complies with the ethical principles of the World Medical Association Declaration of Helsinki [22]. Ethical approval was obtained from the lead Ethics Committee of the Medical Faculty of the University of Duisburg Essen (reference number: 21-9866-BO, date of approval: 15/06/2021). |
| Protocol amendments | 25 | Important protocol modifications changes will be reported to the ethical committee and other relevant parties. |
| Consent or assent | 26a | All participants received written information and sign informed consent forms, which are stored at the rehabilitation centre. |
|  | 26b | No additional consent is provided for collection and use of participant data who withdraw; this study does not involve collecting biological specimens for storage. |
| Confidentiality | 27 | For the statistical analysis, collected data is documented in the rehabilitation center. Pseudonymised data is sent to the Institute for Medical Informatics, Biometry and Epidemiology (IMIBE) for analysis. All employees are familiar with quality-assured data collection, data monitoring and documentation. Record retention periods correspond to the GCP-guidelines. |
| Declaration of interests | 28 | There are no financial and other competing interests for principal investigators for the overall trial and each study site. |
| Access to data | 29 | The final trial dataset belongs to the principal investigator. |
| Ancillary and post-trial care | 30 | Participants are able to contact the research team during the study period as well as after the end of participation. |
| Dissemination policy | 31a | We developed a publication strategy to select appropriate content, formats and audiences. The trial results will be reported to participants and the public in a public-centered research report. Healthcare professionals and the scientific community will be informed via peer-reviewed journal publications and conference proceedings.  According to the guiding principle of the PI’s affiliation, research publications are not influenced or controlled by funders or investigators. We will publish both positive and negative outcomes in an equitable manner. |
|  | 31b | All authors must have contributed substantially to a work's. We will not use professional writers. |
|  | 31c | There are no plans to grant access to full protocol, participant-level dataset or statistical code. |
| Appendices |  |  |
| Informed consent materials | 32 | Each patient received standardized written participant information and an informed consent form. |
| Biological specimens | 33 | This study does not involve collecting and storing biological specimens for storage in the current trials and for future use in ancillary studies. |

*It is strongly recommended that this checklist be read in conjunction with the SPIRIT 2013 Explanation & Elaboration for important clarification on the items. Amendments to the protocol should be tracked and dated. The SPIRIT checklist is copyrighted by the SPIRIT Group under the Creative Commons “[Attribution-NonCommercial-NoDerivs 3.0 Unported](http://www.creativecommons.org/licenses/by-nc-nd/3.0/)” license.


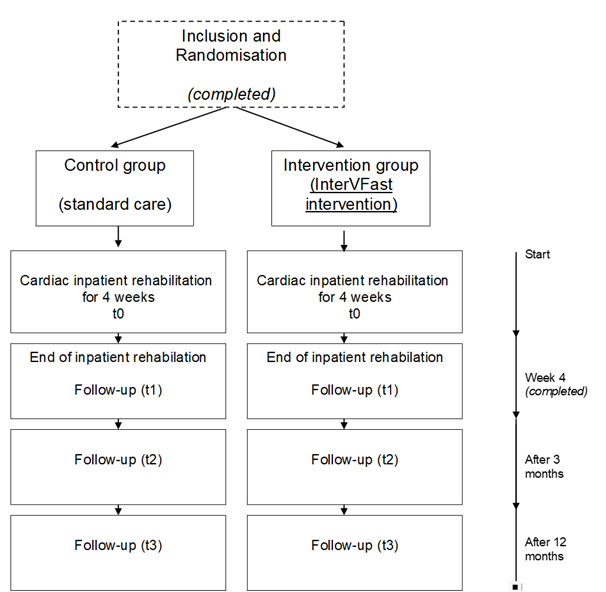


Figure 1_ Time schedule
